# Effects of Fermented Soybean Meal Supplementation on the Growth Performance and Cecal Microbiota Community of Broiler Chickens

**DOI:** 10.3390/ani10061098

**Published:** 2020-06-25

**Authors:** Yang Li, Baozhu Guo, Zhengke Wu, Weiwei Wang, Chong Li, Guohua Liu, Huiyi Cai

**Affiliations:** The Key Laboratory of Feed Biotechnology of Ministry of Agriculture and rural Affairs, National Engineering Research Center of Biological Feed development, Feed Research Institute, Chinese Academy of Agricultural Sciences, No.12 Zhongguancun South Street, Haidian District, Beijing 100081, China; liyang8906@163.com (Y.L.); guobaozhu957@163.com (B.G.); wzk199107@163.com (Z.W.); dbnywzw@163.com (W.W.); lichong@caas.cn (C.L.); liuguohua@caas.cn (G.L.)

**Keywords:** broilers, fermented soybean meal, growth performance, cecal microbiota

## Abstract

**Simple Summary:**

Microbial fermentation is considered an economically viable processing technique to reduce the content of anti-nutritional factors and improve the nutritional quality of soybean meal (SBM). Fermented soybean meal (FSBM) exerts beneficial effects on the growth performance, carcass traits, and meat quality of broilers. However, there is very little information on the effects of FSBM on cecal microbial composition and diversity in broilers. Hence, this trial is conducted to investigate the effects of the partial replacement of SBM with FSBM in the diet on growth performance and cecal microflora of broilers. Replacing 25% of the SBM in the diet of broilers with FSBM promoted average daily gain and feed conversion ratio during the growth and whole phases. Both dietary treatment (25% or 50% of the SBM in the basal diet was replaced with FSBM) influenced the serum immunity, diversity and composition of cecal microbiota in broilers. FSBM supplementation in the diet shifted the cecal microbial community of broilers towards a healthier balance by increasing the abundance of beneficial bacteria and reducing the abundance of potentially harmful bacteria.

**Abstract:**

This study investigated the growth performance, serum immunity, and cecal bacterial microbiota of broilers fed a diet in which soybean meal (SBM) was partially replaced with fermented soybean meal (FSBM) for 36 days. A total of 180 one-day-old male Cobb 500 broilers were randomly divided into three dietary groups (six replicates per group): corn-SBM diet (CC); 25% SBM replaced by FSBM (SC); 50% SBM replaced by FSBM (TC). The average daily gain (ADG) and feed conversion rates (FCR) were higher in SC than CC and TC groups (*p* < 0.05) during the growth (d 22–36) and whole (d 1–36) phases. No significant difference was observed in ADG and average daily feed intake (ADFI) between CC and TC groups during any phases. Dietary treatments increased serum IgA, IgG, and IgM, Chao 1, observed species, and the abundance of the phylum *Fimicutes* but decreased the proportion of *Proteobacteria* (*p* < 0.05). Dietary treatments increased the abundance of the genera *Lachnospiraceae*, *Lachnoclostridium*, *Gastranaerophilales*, and *Lactobacillus* but decreased the abundance of *Escherichia-Shigella* and *Clostridiales* (*p* < 0.05). Spearman’s correlations showed that the abundance of *Gastranaerophilales* was positively correlated with ADG and serum immunity, and the abundance of *Lactobacillus* was strongly positively with IgM. Thus, replacing 25% of SBM with FSBM improves the growth performance and serum immunity of broilers, possibly due to altered cecal microbial composition.

## 1. Introduction

Soybean meal (SBM), a byproduct of oil extraction from soy seed, is the most commonly used plant protein source in the poultry and swine feed industries. However, a variety of anti-nutritional factors (ANFs) present in SBM, such as antigenic proteins, trypsin inhibitors, and oligosaccharides, interfere with digestion and absorption and have negative effects on animal health [1]. Accumulating evidence has demonstrated that microbial fermentation improves the nutritional quality of SBM by reducing the content of ANFs and increasing nutrient bioavailability [2,3,4]. Fermented SBM (FSBM) is produced by the addition of fungal or bacterial microorganisms, including *Lactobacillus plantarum*, *Bacillus subtilis*, *Aspergillus oryzae*, and *Neurospora crassa* [5,6,7]. Zhang et al. [6] showed that 92.36% of glycinin and 88.44% of β-conglycinin was eliminated from SBM, and the amount of trichloroacetic acid-soluble protein (TCA-SP) was increased 4.6-fold after the solid-state fermentation of SBM with *B. subtilis* BS12 for 24 h. A previous study reported that the solid-state fermentation of SBM with *Bacillus amyloliquefaciens* for 48 h degraded soybean macro-molecular proteins to less than 25 kDa and completely decomposed raffinose and stachyose in SBM [2].

Several studies have demonstrated that partial or total replacement of SBM with FSBM improves the growth performance, digestive enzyme activity, and gut morphology of broilers [8,9,10]. Feeding broilers FSBM produced by mixed strains, including *L. plantarum*, *Lactobacillus. acidophilus*, *B. subtilis*, and *A. oryzae*, significantly increased body weight gain and decreased feed conversion ratio (FCR) between d 11 and 24 and d 25 and 42, improved apparent ileal digestibility of crude protein and gross energy, and increased villus height (VH) and villus height to crypt depth (VH: CD) ratios in the duodenum and jejunum [9]. In another study, Jazi et al. [10] reported that dietary FSBM mitigated the growth performance suppression and decrease of VH and VH: CD in the duodenum and jejunum of young broiler chickens challenged by *Salmonella typhimurium* from d 1 to 24. Interestingly, Kim et al. [11] found that feeding broilers 3% FSBM fermented by *Bacillus* alone or in combination with a yeast byproduct in the first 7 days post-hatching significantly increased average daily gain (ADG) and decreased FCR throughout the entire growth phase. In contrast, a diet containing 10% FSBM with complete elimination of glycinin and β-conglycinin did not improve the growth performance of broilers but did increase average feed intake (ADFI) during d 1 to 21 [4]. Therefore, further study of the effects of FSBM produced by different kinds of microorganisms, fermentation times, and processing methods on the growth performance of broilers is needed. 

The intestinal microbiota of animals plays an important role in maintaining intestinal homeostasis and animal health by modulating nutrient digestion, protecting against enteric pathogens, enhancing intestinal immunity, and performing other physiological functions [12]. It was reported that nutrient absorption, feed digestibility, and energy harvest are strongly linked to the gut microbiota and, therefore, animal productivity is influenced by microbial composition and diversity. Furthermore, the gut microbial composition and diversity can be modulated by diet, including feed components and feed additives [13]. FSBM fermented by microorganisms not only increases the nutritional quality of SBM but also provides prebiotics, probiotics, and their metabolites to animals, thereby exerting growth-promoting effects [14]. A previous study in weaning piglets demonstrated that dietary supplementation with FSBM modulated the microbial composition of the colon and feces and increased the abundance of the potentially beneficial bacteria *Lachnospira* and *Lactobacillus* [15]. Xie et al. [16] also found that feeding piglets 15% fresh FSBM altered intestinal bacterial community structure and increased the relative abundance of butyrate-producing bacteria. A recent study using a traditional culture-based approach reported that FSBM supplementation of broiler diets increased the number of lactic acid bacteria, decreased the number of *Coli*-form bacteria and *Clostridium perfringens* in the ileum and cecum [9]. However, the broiler gut microbiota includes hundreds of bacterial species, and it is difficult to cultivate and study the composition, diversity, and structure of intestinal microbiota using traditional culture-based approaches [17]. In recent years, 16s rDNA gene amplicon deep sequencing has been widely applied in a range of studies to investigate microbial composition and diversity and their relationship to the growth performance [17,18] and immunity of animals [15,19,20].

Currently, to the best of our knowledge, there is very little information on the effects of FSBM on cecal microbial composition and diversity in broilers. Therefore, the objective of this study was to investigate the effects of FSBM supplementation on the growth performance, serum immunity, and microflora composition of the cecum of broilers using Quantitative Insights Into Microbial Ecology (QIIME2) and high-throughput sequencing.

## 2. Materials and Methods 

### 2.1. Ethics Statement

Feeding trials were conducted according to the guidelines for animal experiments set out by the National Institute of Animal Health, and all animal procedures were approved by the Chinese Academy of Agricultural Sciences (statement no. AEC-CAAS-20191208).

### 2.2. Preparation of FSBM

SBM was purchased from the Bunge Chia Tai Grain & Oil Co., Ltd. (Tianjin, China). The FSBM used in this study was produced by fermenting SBM with a combination of *B. amyloliquefaciens*, *L. acidophilus*, and *Saccharomyces cerevisiae*. The *B. amyloliquefaciens* and *L. acidophilus* bacterial strains were previously isolated from the intestine of a healthy cow and from silage, respectively. These strains are preserved at the China General Microbiological Culture Collection Center (CGMCC) under preservation numbers CGMCC no. 18230 and no. 14437, respectively. The fungal strain *S. cerevisiae* was stored in our laboratory. The fermentation process was conducted as previously described by Shi et al. [1]. Briefly, each kilogram of SBM fermentation substrate was mixed with 1 L of distilled water containing 6% (v/v) *B. amyloliquefaciens*, 2% (v/v) *L. acidophilus,* and 2% (v/v) *S. cerevisiae.* and fermented at 37 °C for 24 h. After the first stage of fermentation, the fermented mixture was transferred to a plastic bag equipped with a one-way valve (Rou Duoduo Biotechnology Co., Beijing, China) and anaerobically incubated at 37 °C for 24 h. After fermentation, the FSBM was dried at 60 °C for 10 h. The crude protein and dry matter of the SBM or FSBM were measured according to AOAC (2005). The content of TCA-SP in the SBM and FSBM was analyzed using (TCA) according to the China National Standard (GB/T 22492-2008) [21]. The contents of glycinin and β-conglycinin in the SBM and FSBM were tested using competitive enzyme-linked immunosorbent assay (ELISA) kits (Longkefangzhou Bio-Engineering Technology Company, Beijing, China) according to the manufacturer’s protocol. The compositions of the SBM and FSBM are shown in Table 1.

### 2.3. Animals, Management, and Experimental diets

A total of 180 one-day-old Cobb 500 male broilers were purchased from a local commercial hatchery (Beijing Dafa Chia Tai Co., Ltd, Beijing, China) and randomly allotted to three experimental groups consisting of six replicates with 10 birds per replicate. There was no significant difference in the initial bodyweight of broilers in the three groups. Experimental broilers were housed in wire cages in an environmentally controlled house with 16 h of light and were given ad libitum access to feed and fresh water. The room temperature was initially maintained at 33 °C from d 1 to 3 and then gradually decreased according to the age of the broilers until reaching 23 °C at 21 d. In addition, the broilers were vaccinated according to the routine immunization program. The corn-SBM basal diet (CC, control group) was formulated based on the nutrient requirements of broilers recommended by the Ministry of Agriculture and rural Affairs of the People’s Republic of China (NY/T33—2004). In the two dietary treatment groups, either 25% of the SBM (SC group) or 50% of the SBM (TC group) in the corn-SBM basal diet was replaced with FSBM. Diet composition and nutrient levels are shown in Table 2.

### 2.4. Growth Performance and Sample Collection

The bodyweight and feed intake of each replicate were recorded at d 21 and 36, and the ADFI, ADG, and FCR were calculated for the starter (d 1–21), growth (d 22–36), and whole (d 1–36) phases. On d 36 of the trial, one bird from each replicate was randomly selected and euthanized by cervical dislocation. Blood was rapidly collected into serum separation tubes, centrifuged at 3000 rpm for 15 min at 4 °C, and stored at −20 °C until analysis. After dissection, the cecal digesta was collected, and immediately frozen in liquid nitrogen, and stored at −80 °C for DNA extraction.

### 2.5. DNA Extraction and 16S rDNA Polymerase Chain Reaction (PCR) Amplification

Total bacterial genomic DNA was extracted from each cecal digesta sample using an E.Z.N.A. ^®^ Stool DNA Kit (D4015, Omega, Inc., Norcross, GA, USA) according to the manufacturer’s instructions. DNA quantity and quality were determined using a NanoDrop ND-100 spectrophotometer (Thermo Fisher Scientific, Waltham, MA, USA) and agarose gel electrophoresis, respectively. Polymerase chain reaction (PCR) amplification of the V3–V4 region of the bacterial 16S rRNA gene was conducted using the forward primer 341F (5’-CCTACGGGNGGCWGCAG-3’) and the reverse primer 805R (5’-GACTACHVGGGTATCTAATCC-3’). The primers were tagged with 7-bp sample-specific barcodes for multiplex sequencing. The PCR amplification was carried out in a 25-μL reaction mixture containing 25 ng of template DNA, 2.5 μL of each primer, 12.5 μL PCR premix, and PCR-grade water to adjust the volume. The cycling conditions consisted of initial denaturation at 98 °C for 2 min, followed by 32 cycles consisting of denaturation at 98 °C for 10 s, annealing at 54 °C for 30 s, and extension at 72 °C for 45 s, and a final extension at 72 °C for 10 min. PCR amplicons were detected using 2% agarose gel electrophoresis, purified with AMPure XT Beads (Beckman Coulter Genomics, Danvers, MA, USA), and quantified using the Qubit (Invitrogen, Waltham, MA, USA). The amplicon pools were prepared by mixing the equal amount of quantified PCR products, and the size and quantity of the amplicon library were assessed on an Agilent 2100 Bioanalyzer (Agilent, Santa Clara, CA, USA) and with a Library Quantification Kit for Illumina (Kapa Biosciences, Woburn, MA, USA), respectively. The libraries were sequenced on a NovaSeq PE250 platform (Illumina Technologies Co. Ltd, San Diego, CA, USA) [22].

### 2.6. 16S rDNA Gen Sequencing and Bioinformatics Analysis

The 16S rDNA sequencing was carried out on an Illumina NovaSeq platform provided by LC-Bio Technology (Hang Zhou, China) according to the manufacturer’s recommendations. Paired-end reads were assigned to appropriate samples based on unique barcodes and truncated by cutting off the primer and barcode sequence. Paired-end reads were assembled using FLASH software (Columbia, MD, USA) [23]. Using specific filtering conditions, quality filtering was performed on the raw reads to obtain high-quality clean tags based on fqtrim v0.94 (Baltimore, MD, USA). Chimeric sequences were filtered with Vsearch software (v2.3.4, Oslo, Norway). After dereplication using divisive amplicon denoising algorithm 2 (DADA2), we obtained feature tables and feature sequences. Alpha and beta diversity were calculated by random normalization to the same sequences. Then, according to the SILVA (release 132) classifier, feature abundance was normalized using the relative abundance of each sample. Alpha diversity is used to analyze species diversity through five indices, including Shannon, Simpson, Chao1, observed species (OS), Good’s coverage. We calculated these indices for our samples using QIIME 2 (version 2.0, https://library.qiime2.org/). Beta diversity was evaluated to examine the structural variation of microbial communities among treatment groups using UniFrac phylogenetic distances [24,25], and visualized using principal coordinate analysis (PCoA). Differences in the microbiota structure of the groups was assessed by Analysis of similarities (ANOSIM). Linear discrimination analysis (LDA) coupled with effect size (LEfSe) was employed to identify significantly different bacteria among the three groups. Correlations among growth performance, serum immunity, and alterations in the microbiota were evaluated using Spearman’s correlation coefficients.

### 2.7. Statistical Analysis

All statistical analyses were performed using SPSS 19.0 software (SPSS Inc., Chicago, IL, USA). Differences among three groups in growth performance, serum immunity, alpha diversity, and bacterial taxa abundance at the phylum and genus levels were analyzed using one-way analysis of variance (ANOVA) and Duncan’s post-tests. Differences were considered significant if *p* < 0.05 level.

## 3. Results

### 3.1. Effects of FSBM on Growth Performance in Broilers

The effects of FSBM on the growth performance of broilers are presented in Table 3. During the starter phase (d 1–21), the ADG, ADFI, and FCR of broilers were not significantly influenced by the dietary treatments (*p* > 0.05). However, during the growth (d 22–36) and whole phases (d 1–36), although there was no significant difference in ADFI between the CC and SC groups, ADG and FCR were significantly higher in the SC group (*p* < 0.05) compared with the CC group. Strikingly, no significant differences in ADG and ADFI were observed between broilers in the CC and TC groups during the growth or whole phases.

### 3.2. Effects of FSBM on the Serum Immunoglobulin of Broilers

**Figure 1:**
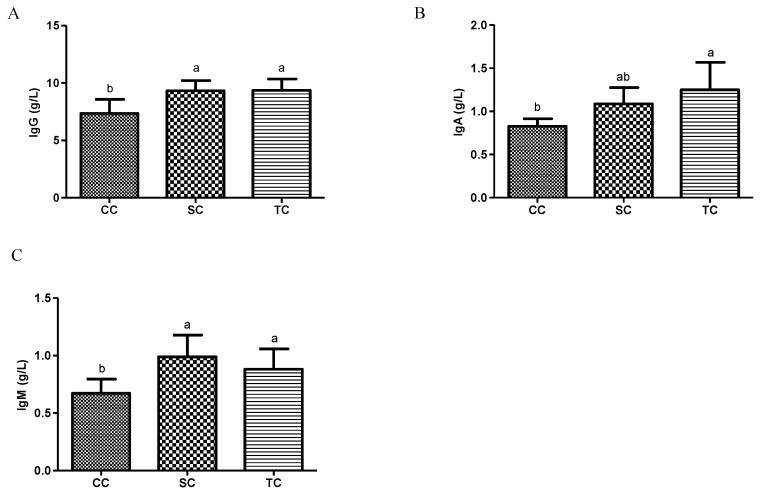
Effects of fermented soybean meal (FSBM) on serum immunoglobulin concentrations in broilers. (**A**) IgM; (**B**) IgG; (**C**) IgA. CC, control group; SC, 25% of soybean meal (SBM) replaced with fermented soybean meal (FSBM); TC, 50% of soybean meal (SBM) replaced with fermented SBM (FSBM). ^a,b^ Means with different letters within columns indicates significant differences at *p* < 0.05.

As shown in Figure 1A–C, the serum concentrations of IgM, IgG, and IgA increased significantly in the SC and TC groups compared to the CC group (*p* < 0.05), but there were no significant differences in serum immunoglobulin levels between the SC and TC groups (*p* > 0.05).

### 3.3. Quality of Sequencing Data

After the quality filtering of the raw reads, the sequencing of 18 samples generated 1,353,140 high-quality clean tags with an average of 75,174 valid tags per sample. A total of 2423 feature tables were obtained after removing replicates and chimeric sequences. A total of 665 feature tables were common among the three groups, and 318, 554, and 517 feature tables were unique to the CC, SC, and TC groups, respectively (Figure S1). Rarefaction curves showed that sufficient sequencing coverage was achieved (Figure S2).

### 3.4. Effects of FSBM on Cecal Microbial Diversity

As shown in Table 4, the alpha diversity of the cecal microbiota of broilers was influenced by dietary treatment. Chao 1 and OS richness estimator of cecal microbial diversity were higher in the SC and TC groups than in the CC group. However, Shannon and Simpson microbial diversity indices were not significantly altered by dietary treatment.

**Figure 2:**
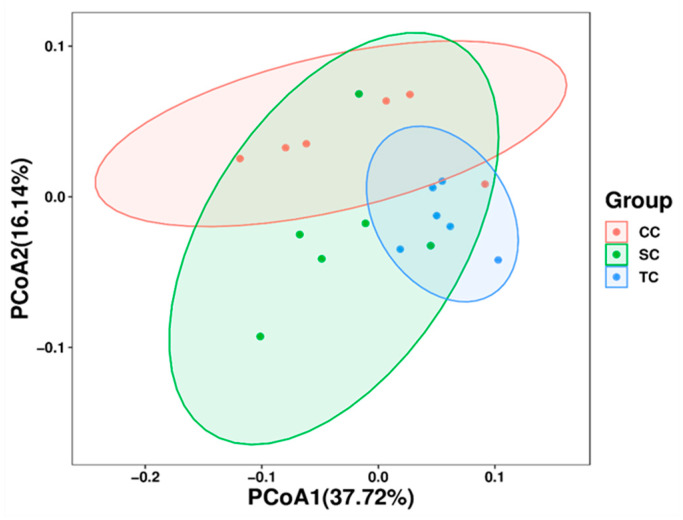
Beta diversity analysis of microbial communities using principal coordinate analysis (PCoA) based on weighted UniFrac distances. CC, control group; SC, 25% of soybean meal (SBM) replaced with fermented SBM (FSBM); TC, 50% of soybean meal (SBM) replaced with fermented SBM (FSBM).

To further analyze the differences in microbial community structure in cecum among control and treatment groups, beta diversity was estimated using PCoA based on weight UniFrac phylogenetic distances. As presented in Figure 2, the results showed that cecal microbiota samples from the CC group clustered together and were clearly separated from those from the SC and TC groups, suggesting that the composition of cecal microbiota differed significantly between control and treatment groups (R = 0.3289, *p* = 0.007).

### 3.5. Effects of FSBM on Cecal Microbial Composition

As shown in Table 5, six phyla were identified in the cecal digesta of broilers: *Firmicutes*, *proteobacteria*, *Bacteroidetes*, *Tenericutes*, *Actinobacteria*, and *Verrucomicrobia*. The predominant phyla were *Firmicutes*, *Proteobacteria*, and *Bacteroidetes*, which accounts for over 98% of all phyla in the cacal digesta of broilers. FSBM led to an increase in the relative abundance of *Firmicutes* in the SC and TC groups compared to the CC group (*p* < 0.05). However, the relative abundance of *Proteobacteria* was lower in the two dietary treatment groups than in the CC group (*p* < 0.05). No significant differences were observed in the relative abundance of *Firmicutes*, *Proteobacteria*, *Bacteroidetes*, *Tenericutes*, or *Actinobacteria* between the SC and TC groups. Interestingly, the phylum *Verrucomicrobia* was present only in the SC and TC groups, and the relative abundance of *Verrucomicrobia* was higher in the SC group than in TC group.

**Figure 3:**
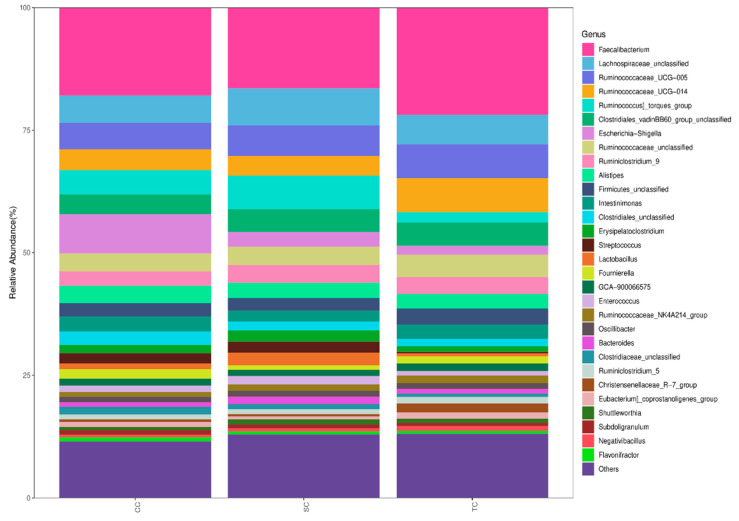
The relative abundance of bacterial genera in the cecal microbiota of broilers in different groups. CC, control group; SC, 25% of soybean meal (SBM) replaced with fermented SBM (FSBM); TC, 50% of soybean meal (SBM) replaced with fermented SBM (FSBM).

We further compared the microbial communities at the genus level. The 30 most predominant genera in the cecal digesta of broilers are presented in Figure 3. The top fifteen dominant genera were *Faecalibacterium*, *Lachnospiraceae*, *Ruminococcaceae_UCG-005*, *Ruminococcaceae_UCG-014*, *Ruminococcus]_torques_group*, *Clostridiales_vadinBB60_group*, *Escherichia-Shigella*, *Ruminococcaceae*, *Ruminiclostridium_9*, *Alistipes*, *Firmicutes_unclassified*, *Intestinimonas*, *Clostridiales*, *Erysipelatoclostridium*, and *Latobacillus*.(Table S1). The dietary treatment did not have a significant effect on the relative abundance of *Faecalibacterium* (*p* > 0.05), the most abundant genus in the cecal microbiota. 

As shown in Table 6, the relative abundance of *Escherichia-Shigella*, *Clostridiales*, and *Anaeroplasma* decreased significantly in the SC and TC groups compared with the CC group (*p* < 0.05). The relative abundance of genera *Lachnospiraceae Lachnoclostridium*, and *Lactobacillus* increased significantly in the SC and TC groups compared to the CC group (*p* < 0.05). The genera *Ruminococcus_torques_group* and *Gastranaerophilalesp* were most abundant in the SC treatment group (*p* < 0.05). Interestingly, the genus *Akkermansia*, which belongs to the phylum *Verrucomicrobia*, was present only in the SC and CC groups, and was higher in the SC group than in the TC group.

**Figure 4:**
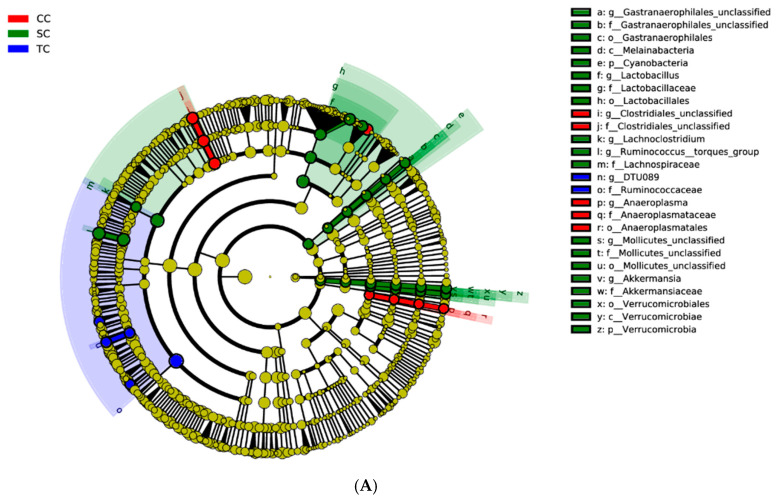

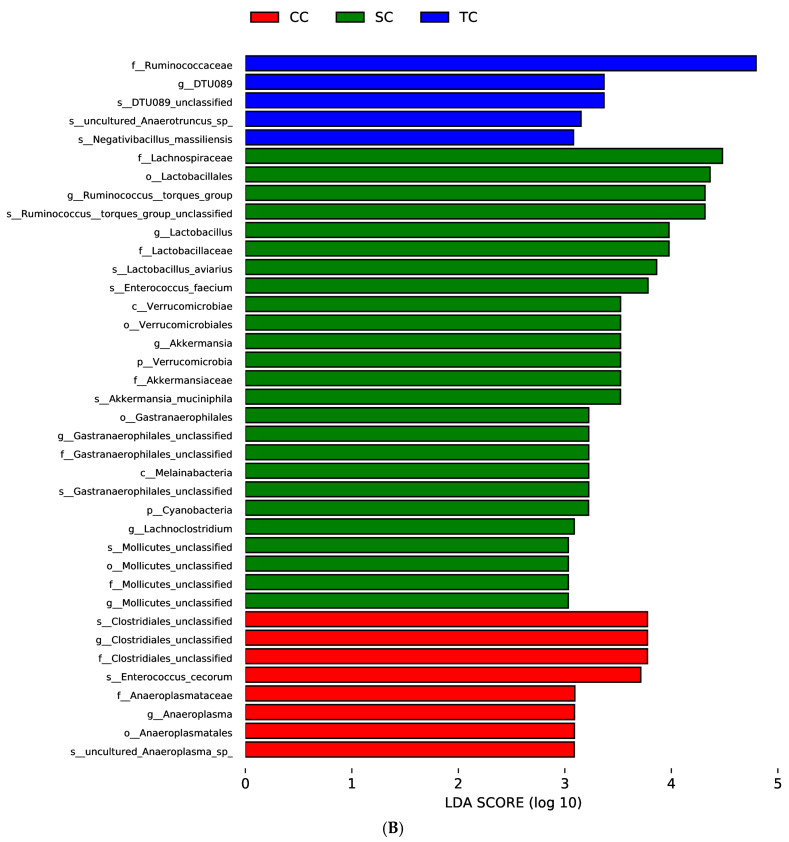
Linear discrimination analysis (LDA) coupled with effect size (LEfSe) analysis of the cecal microbial community of broilers in the CC, SC, and TC groups. (**A**) Cladogram showing microbial species with significant differences among the three treatment groups. Red, green, and blue represent different groups. Species classification at the phylum, class, order, family, and genus levels are displayed from inner to outer layers. The red, green, and blue nodes represent microbial species in the phylogenetic tree that play important roles in the CC, SC, and TC groups, respectively. Yellow nodes represent no significant difference between species. (**B**) Significantly different species with an LDA score greater than the estimated value (default score = 3). The length of the histogram represents the LDA score of different species in the three groups. CC, control group; SC, 25% of soybean meal (SBM) replaced with fermented SBM (FSBM); TC, 50% of SBM replaced with FSBM.

LEfSe was applied to explore the relative richness (*p* < 0.05, LDA > 3) of the cecal microbiota of broilers in the three groups. The results suggest that dietary treatment modified the microbial composition of cecal digesta. The genera *Ruminococcus_torques_group*, *Lactobacillus*, *Akkermansia*, *Gastranaerophilales*, and *Lachnoclostridium* were enriched in the SC group, whereas the microbiota of the CC group was enriched with the genera *Clostridiales* and *Anaeroplasma* (Figure 4).

### 3.6. Correlation Analysis of Altered Cecal Bacteria with Growth Performance and Serum Immunoglobulin

**Figure 5:**
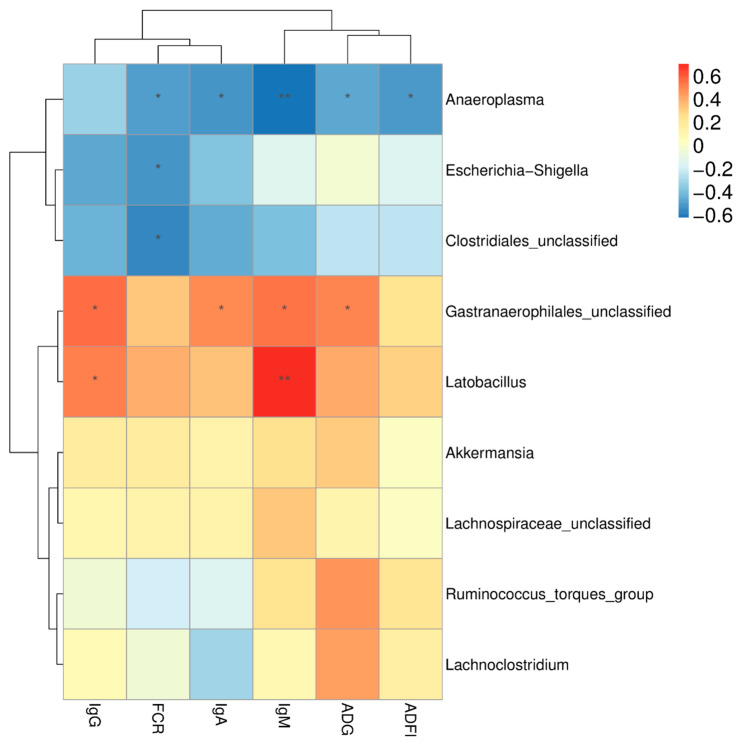
Spearman’s rank correlation analysis between significantly modified microbiota and growth performance and serum immunoglobulin concentrations of broilers. * *p* < 0.05, ** *p* < 0.01. Red represents a positive correlation, and blue represents a negative correlation. ADFI, average daily feed intake; ADG, average daily gain; FCR, feed conversion ratio; IgA, immunoglobulin A; IgM, immunoglobulin M; IgG, immunoglobulin G.

Spearman’s rank correlation analysis was carried out to assess the potential relationship between modifications in cecal microbiota composition and the growth performance and serum immunoglobulin concentrations of broilers (Figure 5). Correlation analysis revealed that the abundance of the genus *Lactobacillus* was strongly positively correlated with IgM and IgG (*p* < 0.05). The abundance of the genus *Gastranaerophilales* was positively correlated with ADG, IgM, and IgA (*p* < 0.05), whereas the genus *Anaeroplasma* was strongly negatively correlated with ADG, IgM, and IgA (*p* < 0.05). In addition, the abundance of the genus *Clostridiales* was negatively correlated with FCR (*p* < 0.05).

## 4. Discussion

SBM is the most commonly used plant protein source in poultry and swine production. However, various ANFs, such as antigenic protein and trypsin inhibitors, limit its wide application in animal feed [26]. A growing number of studies indicate that microbial fermentation is an economically and widely available method to solve the issues of SBM [2,21,27]. In pig production, extensive evidence has shown that FSBM or fermented feed improves growth performance, nutrient digestibility, and immune function [16,19,26,28]. However, to date, information on the effects of FSBM on the growth performance of broilers is limited and inconsistent. In the present study, the results demonstrate that substituting 25% of the SBM in the diets with FSBM (8.18% FSBM addition) significantly increased the ADG and FCR of broilers during both the growth (d 22–36) and whole (d 1–36) phases, compared with those in the control group, but did not affect ADG, ADFI, and FCR during the starter (d 1–22 ) phase. Similarly, Chachaj et al. [29] reported that replacing SBM with 6% FSBM significantly increased bodyweight gain at d 32 and d 40, but did not affect feed intake or FCR. A study of bacteria and fungi-mixed fermented SBM found that supplementing the diets of broiler chickens with 4.5–6.0% wet or dry FSBM had better effects on growth performance than 5% SBM [30]. Feng et al. [8] reported that replacing all SBM in the diets of broiler with FSBM fermented by *A. oryzae* 3.042 significantly improved ADG and ADFI. The enhanced growth performance of broilers fed FSBM in these studies was associated with a reduction in ANFs from SBM, improved intestinal morphology, and increased digestive enzyme activity. However, Guo et al. [31] found that supplementation with 7.5% FSBM, fermented with *B. subtilis*, *L.* spp., and yeasts, did not affect the growth performance of broilers over the entire rearing period. Additionally, feeding broilers 10% FSBM, produced by *B.subtilis* combined with protease, increased higher ADFI but did not improve ADG throughout the entire feeding period [4]. These discrepancies between our observations and previous findings might be attributable to the different types of microorganisms used for fermentation, the various methods used to process FSBM, or the different amounts of FSBM added to the diets. Interestingly, in our study, the ADG and ADFI of broilers in the TC group (50% of SBM in the basal diet replaced with FSBM) did not significantly improve compared with those of the CC group during the whole phase. This result probably relates to the increase in free amino acids that occurs in SBM after fermentation. Previous studies have demonstrated that the absorption of amino acids in the form of free amino acids is much slower and requires more energy than the absorption of oligopeptides [32,33]. Therefore, higher levels of free amino acid in the diets may have affected the growth performance of broilers.

Serum immunoglobulin concentrations are commonly considered to be vital indicators of the humoral immunity of animals, as immunoglobulins are immune-active molecules that play important roles in defending the host against pathogenic viruses and microorganisms [19,34]. IgG, IgM, and IgA are the three major immunoglobulin classes in avian species. In the present study, concentrations of IgG, IgM, and IgA were significantly higher in the two dietary treatment groups, compared with the CC group, suggesting that FSBM enhanced the immune function of broilers. Our results are in agreement with the findings of Zhu et al. [28], who noted a significant increase in serum concentrations of IgG and IgM in weaning piglets fed FSBM. Previous research showed that piglet immunity significantly decreased when β-conglycinin was not adequately deactivated during fermentation [35,36]. Our research revealed an increase in the immune function of broilers in the SC and TC groups that was directly related to the lower levels of glycinin and β-conglycinin in FSBM compared with SBM. Several previous studies observed that the formation of small sized peptides during fermentation were associated with increased immunoglobulin levels in broilers [37,38].

The gut microbiota plays an important role in maintaining the normal physiological structure and function of the intestinal tract. Previous evidence has revealed a close correlation between the growth performance and gut microbiota of different species of animals [17,18,19]. The gut microbial composition is extensively influenced by many factors, such as diet, age, feeding patterns, host genotype, pathogen infections, and feed additives [12]. A previous study indicated that diet has a significant effect (estimated to be 57%, compared with 12% for genetic factors) on the intestinal microbial community structure [39]. Intestinal microbial composition can quickly be altered by changes in dietary components [40]. In the present study, OS and Chao1 richness estimators increased significantly in broilers fed FSBM, indicating greater species richness in the cecal digesta. It has been reported that Chao1 and Shannon indices of alpha diversity were highest in the colon of pigs fed diets containing FSBM [15]. Previous research demonstrated that a highly diverse gut microbiota is more stable and improves the health of animals compared with less diverse microbiota [17,39], as harboring a broad range of bacteria in the intestinal tract enables the flora to better deal with environmental perturbations [41].

Regardless of dietary treatment, *Firmicutes*, *Proteobacteria*, *Tenericutes*, and *Bacteroidetes* were the four pre-dominant bacterial phyla in the cecal microbiota, which is similar to the results of a previous study of broilers [42]. However, replacing SBM in the diets of broilers with FSBM modulated cecal microbiota composition at the phylum level. The relative abundance of *Firmicutes* was significantly increased in the SC and TC groups. *Firmicutes* are associated with the decomposition of polysaccharide and the utilization of energy in the gut owing to their genes encoding non-starch polysaccharides degrading enzymes. Moreover, the abundance of *Firmicutes* and the ratio of *Firmicutes* to *Bacteroides* are often positively correlated with the growth performance of animals [13,43]. Therefore, the increase in *Firmicutes* observed in the dietary treatment groups could at least partially explain the improvement in ADG observed in broilers fed FSBM. Intestinal bacteria belonging to *Proteobacteria* include a wide variety of pathogens such as *Brucella* spp., *Rickettsia* spp., and *Neisseria* spp [44]. In the present study, the relative abundance of *Proteobacteria* was much lower in the two dietary treatment groups than in the CC group, which is consistent with previous research that showed that the addition of FSBM to diets decreased the number of harmful bacteria belonging to the phylum *Proteobacteria,* such as *Escherichia coli* [9]. It has been reported that the phylum *Proteobacteria* has a low abundance in the intestine of healthy humans [44]. However, an increased abundance of *Proteobacteria* has been observed in humans with enteric infections, colorectal cancer, and metabolic syndrome, and may be a microbial signature of dysbiosis in the gut microbiota [45]. In young pigs, undigested dietary proteins in the gastrointestinal tract enter the hindgut and become a substrate of fermentation by some genera belonging to *Proteobacteria*, such as *E. coli*, *Klebsiella* spp., and *Campylobacter* spp [46]. Conversely, these undigested dietary proteins promote the reproduction of these harmful bacteria [47]. Therefore, we speculate that the lower abundance of the phylum *Proteobacteria* that we observed may be related to the improved digestibility of the crude protein in FSBM. On the other hand, the Lactobacillus in FSBM reduces gut pH by the production of organic acids and prevents the colonization of enteropathogens through competitive exclusion, antagonistic activities and bacteriocin production, which is also contributable to decrease the abundance of the phylum *proteobacteria* [48]. Interestingly, we observed that the phylum *Verrucomicrobia* was only present in the two dietary treatment groups. *Verrucomicrobia* is phylogenetically closely related to *Planctomycetes* and *Chlamydiae*, which represent 1–3% of the total microbiota in humans and are negatively correlated with inflammatory bowel disease, Crohn’s disease, and non-obese diabetes [49]. Therefore, the modulation of *Firmicutes*, *Proteobacteria*, and *Verrucomicrobia* may be beneficial to maintain the normal function of the intestine and enhance the growth performance of broilers.

At the genus level, our results showed that the abundance of *Lachnospiraceae*, *Lactobacillus*, and *Lachnoclostridium* increased significantly in broilers fed diets supplemented with FSBM. Studies have shown that *Lachnospiraceae* constitutes one of the major taxonomic groups of the human gut microbiota and is correlated with human health [50,51]. The lower abundance of *Lachnospiraceae* was previously reported in multiple sclerosis and ulcerative colitis patients [51]. In animals, herbivores have a higher abundance of *Lachnospiraceae* than carnivores [50]. All members of *Lachnospiraceae* are anaerobic, fermentative, and chemoorganotrophic and can degrade non-starch polysaccharides and produce acetic acid and butyrate [51]. Butyrate is the primary energy source for host epithelial cell growth, enhances epithelial barrier integrity, and inhibits inflammatory responses [41,52]. In addition, *Lachnospiraceae* is associated with increased FCR and bodyweight gain in broilers [53], which was confirmed in our study. The higher relative abundance of the genus *Lachnospiraceae* may be a result of decomposition of non-starch polysaccharides in SBM during fermentation, as the products of degraded non-starch polysaccharides can be more easily fermented by members of the genus *Lachnospiraceae.* The genus *Lachnoclostridium*—butyric-acid-producing bacteria that have been implicated in the alleviation of gut inflammation—was more abundant in the two dietary treatment groups [54]. *Lactobacillus* is known to have a beneficial effect on the gastrointestinal tract and the growth of broilers and is commonly used as probiotics in animal production [20,42]. *Lactobacillus* promotes the growth performance of animals by protecting the gut from pathogens and improving nutrient and energy extraction by the host [55]. In the present study, the relative abundance of *Lactobacillus* was higher in the two dietary treatment groups than in the control group and was positively correlated with serum IgM and IgG levels. Wang et al. [20] also found that feeding weaning piglets *L. plantarum* PFM 105 increased the serum IgM levels. The genus *Gastranaerophilales* was more abundant in the SC group and was positively correlated with ADG and serum immunity. However, little is known about this microorganism. Ma et al. [56] reported that *Gastranaerophilales* is capable of fermenting a range of sugars (e.g., glucose, starch, and hemicellulose) to produce butyrate in the gut of herbivores. Borsanelli et al. [57] found that the abundance of *Gastranaerophilales* was higher in the mouths of healthy cattle and lower in the mouths of cattle with bovine periodontitis. Notably, *Akkermansia*, the only genus in the phylum *Verrucomicrobiae*, was only present in the two dietary treatment groups. *Akkermansia* has been reported to be a highly specialized bacterium capable of using mucin as its sole carbon and nitrogen source, and of stimulating mucin expression [58]. An increase in *Akkermansia* has been demonstrated to be protective against from inflammatory bowel diseases, metabolic diseases, and neurological disorders [58,59]. Yan et al. [55] observed that *Akkermansia* was more abundant in the cecum of a higher feed efficiency group, and our study similarly demonstrated that the relative abundance of *Akkermansia* was weakly positively correlated with the ADG of broilers. In addition, *Clostridiales* was found to be less abundant in the two dietary treatment groups and strongly negatively correlated with FCR. Another effect of dietary treatment was a significant reduction in *Escherichia-Shigella*, and the decrease in *Escherichia*-*Shigella* was the major contributor to the lower abundance of the phylum *Proteobacteria* compared with control group. The genus *Escherichia–Shigella* includes opportunistic pathogenic bacteria. Previous research has demonstrated that *Escherichia–Shigella* impairs intestinal structure and induces various pro-inflammatory pathways, such as the secretion of virulence factors, resulting in an increased risk of infection and diarrhea in the host [60,61]. The genus *Anaeroplasma* is positively correlated with more severe clinical scores in the animal model of multiple sclerosis, but *Lactobacillus reuteri* treatment reduced its abundance and improved the immunity of animals [62]. Du et al. [63] found that supplementation with *B. amyloliquefaciens* in the diet decreased the abundance of *Anaeroplasma* in growth-retarded beef calves. Our research shows a reduction in the abundance of *Anaeroplasma* in the two dietary treatment groups, suggesting that FSBM supplementation inhibits the reproduction of the potentially harmful bacteria *Anaeroplasma*. The explanation for this requires further investigation in future research.

## 5. Conclusions

In conclusion, replacing 25% of the SBM in the diet of broilers with FSBM promoted ADG and FCR during the growth and whole phases. However, substituting 50% of the SBM in the diet with FSBM did not affect the ADG or ADFI of broilers during any phase. Serum immunoglobulin concentrations improved significantly in broilers fed FSBM compared with those fed SBM. Replacing SBM in the diet with FSBM shifted the cecal microbial community of broilers towards a healthier balance by increasing the abundance of beneficial bacteria and reducing the abundance of potentially harmful bacteria. These findings indicate that FSBM may be a new feed resource to improve growth performance and manipulate the intestinal microbial bacteria of animals.

## Figures and Tables

**Table 1:** Composition of soybean meal (SBM) and fermented soybean meal (FSBM) (air-dry basis, %).

Items	SBM	FSBM
Dry matter, %	89.87	90.18
Crude protein, %	46.64	53.74
TCA-SP, %	2.14	23.14
Glycinin, mg/g	168.35	15.94
β-Conglycinin, mg/g	129.56	20.48
Methione, %	0.47	0.55
Lysine, %	3.06	3.12

**Table 2:** Composition and nutrient levels of experimental diets (air-dry basis, %).

Items	Starter Phase (d 1–21)			Growth Phase(d 22–36)		
	CC	SC	TC	CC	SC	TC
Ingredient						
Corn	57.1	58.02	59.38	59.58	60.65	61.65
Soybean meal	32.64	24.48	16.32	27.91	20.42	13.44
Fermented soybean meal	0	8.18	15.44	0	7.00	13.48
Corn gluten meal	3.00	1.92	1.6	3.72	3.14	2.64
soybean oil	3.00	3.14	3.00	4.20	4.20	4.20
Dicalcium phosphate	1.88	1.88	1.88	1.86	1.86	1.86
DL-methionine	0.22	0.22	0.22	0.20	0.20	0.20
L-Lysine	0.13	0.13	0.13	0.10	0.10	0.10
Limestone	1.15	1.15	1.15	1.15	1.15	1.15
Salt	0.28	0.28	0.28	0.28	0.28	0.28
50% Choline chloride	0.10	0.10	0.10	0.10	0.10	0.10
Premix ^1^	0.50	0.50	0.50	0.50	0.50	0.50
TiO_2_	0	0	0	0.40	0.40	0.40
Total	100	100	100	100	100	100
Calculated nutrient level						
AME (MJ/kg)	12.55	12.55	12.55	12.97	12.97	12.97
Crude protein, %	21.50	21.50	21.50	20.00	20.00	20.00
calcium, %	1.00	1.00	1.00	0.90	0.90	0.90
Available phosphorus, %	0.45	0.45	0.45	0.40	0.40	0.40
Lysine, %	1.15	1.15	1.15	1.00	1.00	1.00
Methionine, %	0.50	0.50	0.50	0.40	0.40	0.40
Methionine + Cystine, %	0.91	0.91	0.91	0.76	0.76	0.76

^1^ The premix provided the following per kg of complete feed: vitamin A, 12,000 IU; vitamin D_3_, 2,000 IU; vitamin E, 20 IU; vitamin K_3_, 2.5 mg; vitamin B_1_, 2 mg; vitamin B_2_, 6 mg; vitamin B_3_, 2 mg; vitamin B_6_, 6 mg; vitamin B_12_, 0.025 mg; D-pantothenic acid, 12 mg; biotin, 0.12 mg; folic acid, 1.25 mg; nicotinic acid, 50 mg; Fe (as ferrous sulfate) 80 mg; Cu (as copper sulfate) 8 mg; Se (as sodium selenite) 0.15 mg; Mn (as manganese sulfate) 100 mg; Zn (as zinc sulfate) 78 mg; I (as potassium iodide) 0.34 mg. CC, control group; SC, 25% of soybean meal (SBM) replaced with fermented SBM (FSBM); TC, 50% of SBM replaced with FSBM.

**Table 3:** Effects of fermented soybean meal (FSBM) on the growth performance of broilers.

Items	CC	SC	TC	SEM	*p*-Value
ADG (g/d/bird)					
1–21 d	45.73	46.79	45.85	0.47	0.64
22–36 d	101.41 ^b^	109.21 ^a^	102.22 ^b^	1.28	0.01
1–36 d	59.67 ^b^	63.73 ^a^	60.10 ^b^	0.63	<0.01
ADFI (g/d/bird)					
1–21d	58.60	61.27	59.35	0.75	0.35
22–36 d	160.35	166.36	163.98	1.47	0.26
1–36 d	100.67	100.46	101.78	0.91	0.21
FCR					
1–21 d	1.29	1.31	1.30	0.01	0.18
22–36 d	1.59 ^b^	1.66 ^a^	1.65 ^a^	0.14	0.08
1–36 d	1.48 ^b^	1.52 ^a^	1.51 ^a^	0.01	0.02

^a,b^ Different superscript letters within a row indicate significant differences at *p* < 0.05. CC, control group; SC, 25% of soybean meal (SBM) replaced with fermented soybean meal (FSBM); TC, 50% of soybean meal (SBM) replaced with fermented SBM (FSBM); ADG, average daily gain; ADFI, average daily feed intake; FCR, feed conversion rates.

**Table 4:** Alpha diversity analysis of the cecal microbiota of broilers.

Items	CC	SC	TC	SEM	*p*-Value
Chao1	462.50 ^b^	551.81 ^a^	518.45 ^ab^	16.01	0.05
OS	458.67 ^b^	540.67 ^a^	507.33 ^ab^	14.12	0.04
Shannon	6.87	7.12	7.18	0.07	0.16
Simpson	0.97	0.98	0.98	<0.01	0.67
Coverage	100	100	100	-	-

^a,b^ Different superscript letters within a row indicate significant difference at *p*< 0.05. CC, control group; SC, 25% of soybean meal (SBM) replaced with fermented soybean meal (FSBM); TC, 50% of soybean meal (SBM) replaced with fermented SBM (FSBM).

**Table 5:** Relative abundance of bacterial phyla in the cecal microbiota of broilers in different groups, %.

Item	Relative Abundance, %				
	CC	SC	TC	SEM	*p*-Value
*Firmicutes*	86.44 ^b^	90.39 ^a^	92.71 ^a^	1.06	0.04
*Proteobacteria*	8.31 ^a^	3.31 ^b^	2.15 ^b^	0.09	0.01
*Bacteroidetes*	4.51	4.62	4.39	0.41	0.98
*Tenericutes*	0.41	0.41	0.33	0.07	0.88
*Actinobacteria*	0.20	0.29	0.21	0.04	0.64
*Verrucomicrobia*	ND ^1^	0.51	0.10	0.14	ND

^a,b^ Different superscript letters within a row indicate a significant difference at *p* < 0.05. CC, control group; SC, 25% of soybean meal (SBM) replaced with fermented SBM (FSBM); TC, 50% of soybean meal (SBM) replaced with fermented SBM (FSBM). ^1^ ND not detected.

**Table 6:** Differentially abundant genera in the cecum of broilers receiving dietary three treatments.

Item	Relative Abundance, %				
	SC	CC	TC	SEM	*p*-Value
*Lachnospiraceae*	5.52 ^b^	7.58 ^a^	6.22 ^ab^	0.36	0.04
*Ruminococcus_torques_group*	4.95 ^b^	6.88 ^a^	2.11 ^c^	0.69	<0.01
*Clostridiales*	2.74 ^a^	1.8 ^b^	1.46 ^b^	0.26	0.02
*Escherichia-Shigella*	6.86 ^a^	2.99 ^b^	1.82 ^b^	0.85	0.03
*Lactobacillus*	0.89 ^c^	2.09 ^a^	1.35 ^b^	0.20	0.04
*Gastranaerophilales*	0.10 ^b^	0.42 ^a^	0.20 ^b^	0.05	0.01
*Akkermansia*	ND ^1^	0.51 ^a^	0.06 ^b^	0.15	0.36
*Lachnoclostridium*	0.20 ^c^	0.46 ^a^	0.37 ^b^	0.04	0.02
*Anaeroplasma*	0.23 ^a^	0.01 ^b^	0.06 ^b^	0.03	<0.01

^a,b,c^ Different superscript letters within a row indicate significant difference at *p* < 0.05. CC, control group; SC, 25% of soybean meal (SBM) replaced with fermented SBM (FSBM); TC, 50% of soybean meal (SBM) replaced with fermented SBM (FSBM). ^1^ ND not detected.

## Supplementary Materials

The following are available online at https://www.mdpi.com/2076-2615/10/6/1098/s1, Figure S1: Venn diagram of feature tables in the cecal microbiota of broilers in different groups; Figure S2: Rarefaction curve of the microbial species; Table S1: Relative abundance of the top fifteen dominant genera in the cecal microbiota of broilers in different groups, %.
