# Supplementary material for: Effects of Fermented Soybean Meal Supplementation on the Growth Performance and Cecal Microbiota Community of Broiler Chickens
Source: Animals (Basel). 2020 Jun 25;10(6):1098. doi: 10.3390/ani10061098 (PMC7341335; doi:10.3390/ani10061098)
Supplement: Supplementary file 1 [file animals-10-01098-s001.pdf]

animals

Supplementary Materials: Effects of Fermented Soybean Meal Supplementation on the Growth Performance and Cecal Microbiota Community of Broiler Chickens

Yang Li, Baozhu Guo, Zhengke Wu, Weiwei Wang, Chong Li, Guohua Liu and Huiyi Cai

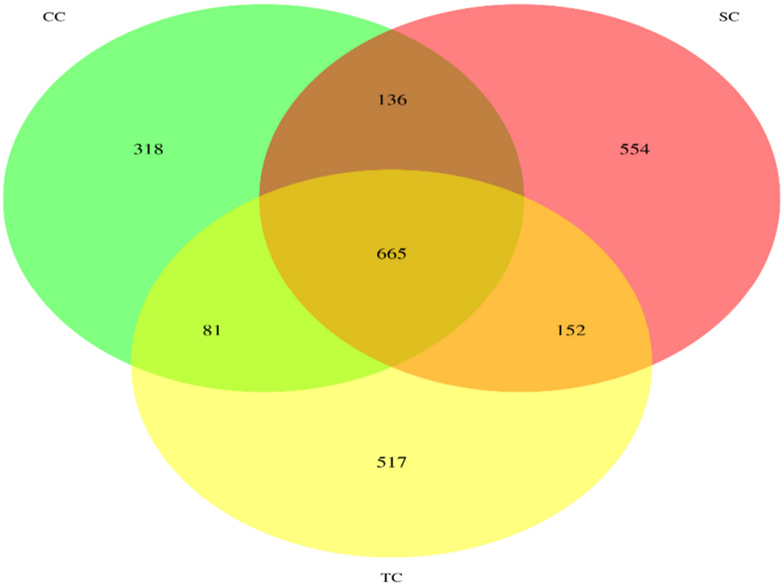

Figure S1. Venn diagram of feature tables in the cecal microbiota of broilers in different groups. CC, control group; SC, 25% of soybean meal (SBM) replaced with fermented SBM (FSBM); TC, 50% of soybean meal (SBM) replaced with fermented SBM (FSBM).

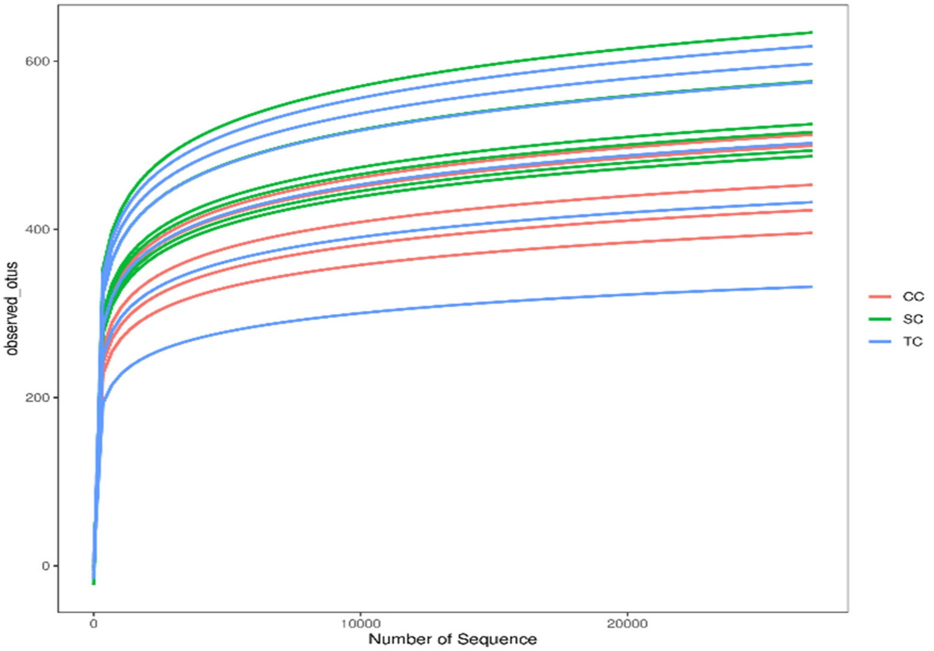

Figure S2. Rarefaction curve of the microbial species. CC, control group; SC, 25% of soybean meal (SBM) replaced with fermented SBM (FSBM); TC, 50% of soybean meal (SBM) replaced with fermented SBM (FSBM).

Table S1. Relative abundance of the top fifteen dominant genera in the cecal microbiota of broilers in different groups.

**Items**	**Relative Abundance, %**				
	**CC**	**SC**	**TC**	**SEM**	***p*-Value**
Faecalibacterium	18.11	17.9	21.88	1.64	0.59
Lachnospiraceae	5.52 [b]	7.58 [a]	6.22 [ab]	0.36	0.04
Ruminococcaceae_UCG-005	5.38	6.25	6.94	0.78	0.74
Ruminococcaceae_UCG-014	4.25	4.47	6.92	0.61	0.23
Ruminococcus]_torques_group	4.95 [b]	6.88 [a]	2.11[c]	0.69	<0.01
Clostridiales_vadinBB60_group	4.02	4.64	4.77	0.48	0.81
Escherichia-Shigella	6.86 [a]	2.99 [b]	1.82 [b]	0.85	0.03
Ruminococcaceae	3.7	3.74	4.54	0.26	0.36
Ruminiclostridium_9	2.91	3.63	3.48	0.22	0.38
Alistipes	3.59	3.17	2.97	0.32	0.76
Firmicutes_unclassified	2.68	2.53	3.24	0.21	0.34
Intestinimonas	3.06	2.25	2.96	0.39	0.69
Clostridiales	2.74 [a]	1.80 [b]	1.46 [b]	0.26	0.02
Erysipelatoclostridium	1.69	2.27	1.15	0.22	0.36
Latobacillus	0.89 [c]	2.09 [a]	1.35 [b]	0.20	0.04

[a,b,c] Different superscript letters within a row indicate significant difference at $p < 0.05$. CC, control group; SC, 25% of soybean meal (SBM) replaced with fermented SBM (FSBM); TC, 50% of soybean meal (SBM) replaced with fermented SBM (FSBM).

© 2020 by the authors. Submitted for possible open access publication under the terms and conditions of the Creative Commons Attribution (CC BY) license (http://creativecommons.org/licenses/by/4.0/).
